# New Approaches in Vital Pulp Therapy in Permanent Teeth 

**Published:** 2013-12-24

**Authors:** Jamileh Ghoddusi, Maryam Forghani, Iman Parisay

**Affiliations:** a *Dental Research Center and Department of Endodontics, Dental School, Mashhad University of Medical Sciences, Mashhad, Iran; *; b *Dental Material Research Center and Department of Pediatric Dentistry, Dental School, Mashhad University of Medical Sciences, Mashhad, Iran*

**Keywords:** Cvek Pulpotomy, Partial Pulpotomy, Permanent Teeth, Pulp Capping, Vital Pulp Therapy

## Abstract

Vitality of dental pulp is essential for long-term tooth survival. The aim of vital pulp therapy is to maintain healthy pulp tissue by eliminating bacteria from the dentin-pulp complex. There are several different treatment options for vital pulp therapy in extensively decayed or traumatized teeth. Pulp capping or pulpotomy procedures rely upon an accurate assessment of the pulp status, and careful management of the remaining pulp tissue. The purpose of this review is to provide an overview of new approaches in vital pulp therapy in permanent teeth.

## Introduction

Vital pulp therapy (VPT) is defined as a treatment which aims to preserve and maintain pulp tissue that has been compromised but not destroyed by caries, trauma, or restorative procedures in a healthy state. This is particularly important in the young adult tooth with incomplete apical root development. It has been recommended that VPT should be performed only in young patients because of the high healing capacity of pulp tissue compared to older patients [1, 2]. Since the evidence regarding the effect of the patients’ age and the status of the root apex on the outcome of VPT did not indicate that this treatment could not be performed successfully in old patients, and based on the premise of innate capacity of pulp tissue for repair in the absence of microbial contamination, preservation of the pulpally involved permanent tooth is also considered [3].

Another important benefit for preservation of vital pulp is the protective resistance to mastication forces compared with a root-canal-filled tooth [4]. It is reported that the survival rate of endodontically treated teeth is not as good as vital teeth especially in molars [5]. Therefore, the vital pulp should be preserved if possible. 

One of the most important issues in VPT is the status of the pulp tissue. The traditional school of thought is that VPT should only be carried out in teeth with signs and symptoms of reversible pulpitis [6]. The problem is how we can accurately assess the status of the pulp. The clinical signs and symptoms such as sensibility and pain testing do not precisely reflect the pulp condition [7-9]. Furthermore, several studies have reported successful treatment outcome in vital teeth with curiously exposed pulp with signs and symptoms of irreversible pulpitis and periapical lesions [10-13]. The degree of pulpal bleeding may be a better indicator of pulpal inflammatory status  [10]. Increased bleeding on exposure site that is difficult to stop, suggest that the inflammatory response extends deeper into the pulp tissue and the treatment procedure should be modified, for example by shifting from direct pulp cap to partial pulpotomy.

Additionally, other factors may affect the success of VPT. The presence of an adequate* blood supply* is required for the maintenance of the pulp vitality [14]. In addition, the presence of a *healthy periodontium* is necessary for success of VPT, and teeth with moderate to severe periodontal disease are not suitable candidates for the treatment [15]. 

Suitable candidates for VPT include teeth in which an appropriate *coronal seal* can be provided. The prognosis of VPT is significantly reduced in cases with inadequate coronal seal and subsequent bacterial microleakage [16].

*Control of hemorrhage* is also necessary for the success of VPT [10]. Various options are available for the achievement of pulp hemostasis such as mechanical pressure using a sterile cotton pellet which may be soaked in sterile water or saline. In addition, disinfection protocols should be also followed as a main principle [17]. Sodium hypochlorite (NaOCl) has been suggested as an agent in VPT that can control hemorrhage, remove the coagulum and dentin chips, disinfect the cavity interface, and aid in formation of dentinal bridge [18]. Another important factor in the success of VPT is a *suitable dressing* material. Pulp covering material should be biocompatible, noncytotoxic, and antibacterial [19].

This article aims to review VPT techniques, namely pulp capping and pulpotomy, in permanent teeth. 

**Indirect pulp capping**

Caries is the result of an ecologic shift within the dental biofilm to acidogenic and aciduric bacterial biofilm. So it seems that with reduction of bacterial number and activity, the ecologic and metabolic balance within the biofilm will be re-shifted, thus remineralization is promoted and the caries lesions is expected to be arrested. It has been shown that after sealing the cavity, carious dentin contains a decreasing number of viable bacteria over time [20, 21].

Indirect pulp capping (IPC) is defined as a procedure in which carious dentin closest to the pulp, is preserved to avoid pulp exposure and is covered with a biocompatible material [22]. Recent studies showed high survival rate (more than 90%) for permanent teeth after performing IPC, without adverse clinical symptoms or pathologic signs on radiographs [20, 23], which were not inferior to those of pulpectomy treatment [21, 24].

This treatment method is intended to protect *primary* odontoblasts and promote *reactionary dentin* formation at the pulp–dentine junction [23, 25]. However, some primary odontoblasts may be destroyed depending on the severity of carious involvement of the dentin-pulp complex and reparative dentin is formed in conjunction with reactionary dentin [23]. An important function of the bioactive lining material is to stimulate the odontoblasts to form reactionary and reparative dentin and promote remineralization of existing dentine, thus encouraging the dentin-pulp complex [26].

Traditionally*, Calcium hydroxide (CH)* has been the material of choice in indirect pulp capping because of its alkaline pH and biocompatible properties that induces pulpo-dentin remineralization. However, since concerns exist regarding its long-term solubility and lack of adhesion to dentin [27], some adhesive materials such as resin modified glass ionomer cements (RM-GIC) have been also suggested.

In a systemic review, Mickenautsch *et al*., evaluated the pulp response to CH and RM-GIC in deep cavities, and found no significant differences between the two materials [28]. Also Hayashi *et al*. mentioned that other materials such as antimicrobials and polycarboxylate cement combined with tannin-fluoride preparation are suitable as liner, since all these materials can reduce cariogenic bacteria and promote remineralization, as effectively as CH [29].

Recently in a clinical trial, *mineral trioxide aggregate (MTA)* has been used as a lining material and was compared with CH [30]. After 6 months, the success rate and the average thickness of newly formed dentin were similar in two groups. However, additional investigations are needed to assess the effectiveness of MTA for IPC.

There are two treatment approaches for performing IPC: *incomplete caries removal with no re-entry* and *stepwise *or* two-step excavation* approach. In stepwise caries treatment, carious dentin in proximity to the pulp remains at the first step and in a second visit some month later, a re-entry procedure is performed. In this step, complete removal of all curious tissue and a definitive restoration are provided. In step-wise approach, removal of carious dentin during re-entry, must be carried out with caution to avoid pulp exposure, since the remaining carious dentin may have become harder, but the thickness of the remaining dentin may be unchanged [29].

Based on the results of a recent systematic review, incomplete caries removal reduces the risk of pulpal exposure and postoperative pulpal symptoms compared with step wise caries removal [31]. However, the amount of dentin that can be left in the cavity was not sufficiently clear. The consensus on this issue is to remove peripheral caries dentin completely to achieve firm marginal adhesion, to remove as much of the caries adjacent to the pulp as possible, and avoid pulp exposure [29]. 

Other questions that still remain are: does residual caries affect physical properties of restored teeth and could the lower bonding strength of resins to carious dentin be overcome by the use of other materials or techniques? To answer these questions and before definitive conclusion can be drawn, further calibrated, multi-centered randomized studies is required.

**Direct pulp capping**

Direct pulp capping (DPC) is defined as the treatment of a mechanical or traumatic vital pulp exposure by sealing the pulpal wound with a biomaterial placed directly on exposed pulp to facilitate formation of reparative dentin and maintenance of the vital pulp (American Association of Endodontists guideline, 2003). As primary odontoblasts are destroyed at the site of pulp exposure and inflammation is initiated, recruitment and differentiation of progenitor/stem cells in the underlying uninfected vital pulp is required to produce reparative dentin [23].

Growth factors such as transforming growth factor (TGF) family released from the dentin matrix and extracellular matrix molecules can induce the differentiation of progenitor/stem cells into odontoblast-like cells. Pulp capping materials such as CH and MTA induce reparative dentin formation by causing the release of growth factors from the dentin matrix [32]. Pulp covering agents in DPC are discussed in detail later.

It has been recommended that DPC should be performed only in teeth with a recent mechanical or traumatic pulp exposure [6]. Recently, VPT in permanent teeth with curiously exposed pulp has been reviewed [3]. The success rate of DPC was 87.5 to 95.4% depending on follow up duration that is not inferior compared to values reported by the literature on treatment outcomes after iatrogenic pulp exposure (ranging from 70 to 98%) [4].

It seems that microorganisms are the key factor in outcome of DPC [4, 6, 33]. Unfavorable outcomes can result from infection due to either remaining bacteria, or new bacteria penetrating from filling margins. Thus, beside the use of rubber dam and aseptic treatment condition the cavity should be restored immediately with a bacteria-tight restoration.

Some studies have evaluated the effect of age, sex, teeth, presence of spontaneous pain, size of exposure and bleeding on the success rate of treatment. Mentioned factors had no significant influence on the success rate except that less bleeding increases the healing of pulp tissues [10].

Asgary and Ahmadyar published a proposed hypothesis in order to achieve improved treatment outcomes of DPC for cariously exposed pulp using *miniature pulpotomy procedure *(MPP) [34]. They claimed that MPP will result in improved treatment outcomes of DPC by improved maintenance of a clean surgical pulp wound; removal of infected dentin chips/damaged pulp tissue specially injured odontoblast cells; improved proximity/interaction of pulp covering agents to undifferentiated mesenchymal/stem cells; better control of bleeding; and creating an improved seal using pulp covering agents (PCAs). Further appropriate clinical trials for approving this hypothesis are recommended.

**Pulpotomy**

Pulpotomy is carried out with two treatment approaches: partial and full pulpotomy.

***Partial pulpotomy***

Partial or Cvek pulpotomy is defined as “the surgical removal of a small portion of the coronal pulp tissue to preserve the remaining coronal and radicular pulp” (American Association of Endodontists guideline, 2003). The inflamed tissue is removed to the level of healthy coronal pulp tissue. Tooth responsiveness to electric pulp tests has been reported in many cases of partial pulpotomy because of preserving the vitality of coronal pulp tissue [35, 36].

Partial pulpotomy has some advantages compared to direct pulp capping such as: removal of the superficially inflamed pulp tissue and providing space for the dressing material which gives the opportunity to seal the cavity. The reported success rate for partial pulpotomy is 93-96% [37, 38].

***Full pulpotomy***

This procedure is defined as “the surgical removal of the entire coronal portion of the vital pulp to preserve the vitality of the remaining radicular portion” (American Association of Endodontists Guideline, 2003). This treatment approach is indicated when it is predicted that the inflammation of the pulp tissue has extended to deep levels of the coronal pulp. The cellular and molecular mechanism of dentin bridge formation after pulpotomy is similar to reparative dentinogenesis following direct pulp capping [25]. After the removal of the coronal pulp, hemostasis must be achieved and a (bio)material is placed over the remaining pulp tissue.

**Dressing materials or pulp covering agents**

***Calcium hydroxide***

The introduction of calcium hydroxide (CH) products played an important role in the development of VPT. However, despite its long history, long-term study outcomes have been variable [33, 39]. Some advantages of CH are antimicrobial characteristics owing to its high alkaline pH and the irritation of pulp tissue that stimulates pulpal defense and repair [40]. Furthermore, its ability to extract growth factors and bioactive dentin matrix components from mineralized dentin can induce dentin regeneration at the site of pulpal exposure [41].

Conversely CH is extremely toxic to cells in tissue culture [42]. It can degrade and dissolve beneath restorations and dentin bridges beneath CH showed porosity and tunnel defects [42, 43]. The disintegration of CH under restorations associated with porosity in the dentinal bridge can provide a pathway for microleakage.

***Resin modified glass ionomers (RMGIs)***

RMGIs have been successful as indirect pulp capping agent even in cavities with minimal remaining dentin thickness [44]. This may be due to their capacity to bond to the dentin and their antimicrobial effect [45]. Contrary to these useful properties, poor responses have been reported in direct pulp capping of human teeth with RMGIs. Pulp tissues that were capped with Vitrebond (3M ESPE, St Paul, Minn., USA) exhibited moderate to intense inflammatory responses, including large necrotic zone, and lack of dentin bridge formation. Thus, the application of RMGIs directly on the pulp tissue is not recommended [46].

***Adhesive resins***

Recently available self-etching adhesive systems as pulp capping material resulted in unresolved inflammatory responses and minimal pulp tissue repair [47, 48]. Many of the resin components in dentin adhesives are vasorelaxant [49] and promote bleeding after hemostasis has been achieved with hemostatic agents. The plasma extravasation may compromise the adhesive polymerization and lead to an increase in their cytotoxicity. Furthermore, the presence of resin particles observed in the pulp seemed to be a trigger in simulating the inflammation and foreign body reactions. The lack of the reparative bridge formation maybe due to this unresolved inflammation [50]. It seems that the adhesive resins are unacceptable as pulp capping agents.

***Mineral trioxide aggregate***

This biomaterial was recommended initially as a root-end filling material in surgical endodontic treatment. Since early scientific observation demonstrating favorable biologic responses to the material, its other usages such as dressing material in vital pulp therapy have been under investigation. Research on this novel endodontic biomaterial had a rapid positive trend especially during the last 5 years [51]. MTA has been shown to induce the recruitment and proliferation of undifferentiated cells and their differentiation to odontoblast-like cells [52]. Dental pulp cells demonstrated higher activation levels in direct contact with MTA that could lead to faster and more predictable formation of dentinal bridge and more effective pulpal repair [53] Histologically, the calcified bridge formed in contact with MTA is thicker with less pulpal inflammation compared to CH [54, 55]. With respect to its success rate, MTA provided a superior performance compared with CH [56]. MTA pulpotomy of symptomatic permanent teeth was also evaluated [57, 58]; none of the patients experienced pain after pulpotomy. Histologic evaluation revealed that all samples had formed dentin bridges and the pulps were vital and almost free of inflammation [57].

However, MTA has some drawbacks such as difficult handling properties, long setting time, high cost, and the discoloration potential of the tooth [59]. As a result of these limitations, a variety of materials have been proposed as candidates for VPT.

***Bioceramics***

Recently, EndoSequence Root Repair Material (ERRM, Brassler, Savannah, GA, USA), BioAggregate (Verio Dental Co, Vancouver, Canada), Biodentin (Septodont, Saint-Maur-des-Fosses, France) and many other bioceramic-based products have been introduced which can be used with the same applications as MTA. Cytotoxicity of ERRM was similar to that of MTA [60]. Biodentin and MTA had also a similar efficacy in pulp-capping treatment [61]. However, further studies are necessary to evaluate these materials in vital pulp therapy.

***Calcium enriched mixture***

Calcium enriched mixture (CEM) cement (Yektazist Dandan, Tehran, Iran) was introduced to dentistry as an endodontic filling biomaterial (USPTO number: 7,942,961). The major components of the cement powder are calcium oxide (CaO), sulfur trioxide (SO_3_), phosphorous pentoxide (P_2_O_5_), and silicon dioxide (SiO_2_). The physical properties of this biomaterial, such as flow, film thickness, and primary setting time are favorable [62], and its clinical applications are similar to those of MTA [63-66].

Several animal studies have shown that in various forms of VPT treatments, the induction of dentin bridge formation in teeth treated with CEM was comparable to that of MTA and superior to CH [67, 68]. Studies of complete pulpotomy treatment using CEM, MTA, and CH have shown that compared to CH, samples in the CEM group exhibited lower inflammation, improved quality/thickness of calcified bridge, superior pulp vitality status, and morphology of odontoblast cells. However, no significant differences were identified in comparison to MTA [68].

DPC outcomes of prospective randomized clinical trials carried out on 32 permanent premolar teeth that were orthodontically planned for extraction have shown that under immunohistochemical examinations, thickness of dentinal bridge and pulp inflammation beneath CEM was comparable to MTA at various time intervals [69].

Indirect pulp therapy with CEM cement on a symptomatic permanent molar also showed favorable results [70]. A randomized clinical trial study on permanent molars with open apices which presented with extensive caries and signs of reversible/irreversible pulpitis was carried out on 51 subjects. Results of a one-year follow-up indicated that complete pulpotomy of the teeth using MTA and CEM were 100% successful [71].

CEM pulpotomy of symptomatic permanent teeth was also evaluated [72]. In a case series study of 12 permanent mature molars with irreversible pulpitis, CEM was used for pulpotomy, and resulted in complete success at a 16-month follow-up. It was also shown that to enable improved regeneration, the pulp-dentin complex had isolated itself by forming a calcified bridge [73]. In a multicenter randomized clinical trial in 23 dental centers linked to five medical universities in Iran, pulpotomy treatment of mature permanent molars diagnosed with irreversible pulpitis was performed using CEM and MTA. The results of this trial showed that pulpotomy treatment carried out by trained dentists can result in successful control of pain [74] and indicated high clinical/radiographic success rates (>92%) during a follow-up after one and two years [70, 75, 76].

***Other bioactive materials for VPT***

Other materials have been also evaluated as a pulp capping agent such as *Enamel Matrix Derivative *(EMD) and *Propolis*. The major constituent of EMD is amelogenin that during odontogenesis is secreted from preamelobasts to differentiating odontoblasts in the dental papilla.

A study comparing the effect of *EMD* and CH as a pulp capping agent demonstrated that CH-treated teeth had less inflammation and more dentine bridge formation than those in EMD-treated teeth [37]. But in the other studies the amount of reparative dentin formed in the EMD-treated teeth was significantly higher than in the CH-treated specimens [77, 78]. The effect of combination of capping materials with EMD has also been evaluated [79-81]. EMD would increase the quality of capping by increasing biocompatibility of capping agents [80]. Furthermore, EMD provide supplemental molecular crosstalk for the formation of dentin-like reparative tissue [81].

As a natural product, Propolis has demonstrated potent antimicrobial and anti-inflammatory properties. Propolis has shown to inhibit synthesis of prostaglandins and supports the immune system by promoting phagocytic activities, stimulating cellular immunity and augmenting healing effects. Additionally, it contains some elements (*i.e.* zinc and iron) that are important for the collagen synthesis [82, 83].

One study demonstrated the pulp response to Propolis was comparable to MTA and better than Dycal [84]. The advantages of this material over CH were confirmed by another investigation. In addition to producing no pulpal inflammation, Propolis induces the production of high quality tubular dentin [85]. However, in order to reach a definite conclusion about these materials, further investigations are needed.

## Discussion

In last decades the concept of self-strangulation during pulpal inflammation has been changed [86]. According to this theory that gained support from the work by Van Hassel, an increase in blood pressure in the pulp tissue with low compliance environment leads to compression of venules that would then result in localized ischemia and necrosis [87]. Several studies have shown that inflammation increases the interstitial blood pressure in the dental pulp. However, experiments have also shown that the pressure increase during pulpitis may be a localized phenomenon, which does not necessarily involve the entire pulp [87, 88]. A few millimeters far from the inflamed area, the tissue pressure might be less and is only slightly higher than in normal non-inflamed pulps. Accordingly, in spite of vasodilatation and increased vessel permeability, the pulpal volume during pulpitis may be remained relatively constant so that the tissue pressure does not rise to a level that will cause vessel compression and total necrosis. Thus, coronal pulpitis may persist without spreading to the root pulp, however, in case of severe persistent irritants, the increased tissue pressure may spread in an apical direction and cause total pulp necrosis [89].

Several histologic studies demonstrated that the cariously exposed vital pulp was not always completely infected, depending on the duration and severity of the carious lesion [8, 9, 90]. Occasionally, the inflammation was localized adjacent to the carious lesion, not spreading to the whole coronal and radicular pulp [90]. If infected tissue is removed, the conservation of the remaining healthy pulp is possible. When encountering cariously exposed pulp, assessing the condition of the pulp, which plays a critical role in the success or failure of VPT, seems difficult if not impossible. There is no reliable tool to help evaluate how far the inflammation has progressed into the pulp. Matsuo *et al.* suggested observing the degree of pulpal bleeding rather than relying on preoperative clinical signs and symptoms [10].

It has been recommended that VPT should be performed only in young patients [1, 2]. However, patients with age ranging from 6–70 years have been successfully treated with VPT [10, 33, 56, 91]. It puts an emphasis the high healing capacity of pulp tissue in both young and old patients after the removal of the etiologic factors. It is likely that the complete removal of the inflamed pulp rather than the status of the root apex is the critical point; recent evidence indicates that VPT could also be performed successfully in old patients [3].

Dentists are traditionally taught that when a patient complains of spontaneous pain or prolonged pain upon cold stimulus, the diagnosis would be irreversible pulpitis and complex expensive pulpectomy is indicated. There are some researches showing that the vital pulp of permanent mature molars with clinical signs of irreversible pulpitis were successfully treated with VPT using appropriate biomaterials (10-13, 68). Therefore, there may be an indication to reconsider the classification of dental pulp diseases, thus create a paradigm shift in endodontic treatments. 

*Control of hemorrhage* is an important key to enhance the success rate of vital pulp therapy. Hemorrhage of exposed dental pulp tissue is in part due to the inflammatory response of the pulp to bacteria and their by-products from carious dentin. Placement of material over a bleeding pulp may criticize the maintenance of vital pulp tissue. Using the hemostatic agents has been recommended over the exposure to halt hemorrhage and allow capping materials to be placed in a relatively dry environment. Sodium hypochlorite has been suggested to remove the coagulum, control hemorrhage, remove dentin chips and aid formation of dentin bridge [92]. Two other important factors in predicting pulpal responses to VPT are the *sealing* ability and the *non-toxicity* of material. However, bacterial contamination is believed to be the main factor [18, 93].

## Conclusion

To improve the success rate of vital pulp therapy, a consensus amid literature should be achieved to recognize various VPT progresses and to incorporate the latest available information into clinical practice and teaching. Further research and clinical trials are also needed to develop case selection guideline, treatment approaches, and materials needed to maximize clinical success. Hopefully in the near future with more knowledge about the biology of the pulp, we can do vital pulp therapy with more predictable outcomes.
